# Slow oocyte freezing and thawing in couples with no sperm or an insufficient number of sperm on the day of *in vitro *fertilization

**DOI:** 10.1186/1477-7827-9-19

**Published:** 2011-02-02

**Authors:** Irma Virant-Klun, Liljana Bacer-Kermavner, Tomaz Tomazevic, Eda Vrtacnik-Bokal

**Affiliations:** 1 Reproductive Unit, Department of Obstetrics and Gynaecology, University Medical Center Ljubljana, Slovenia

## Abstract

**Background:**

The clinical results of *in vitro *fertilization of slowly frozen-thawed oocytes are known to be significantly worse than those obtained by fresh oocytes. Little is known about the factors affecting the clinical outcome of frozen-thawed oocytes. The aim of this retrospective study was to explore the role of oocyte cryopreservation in the group of patients with no available sperm on the day of *in vitro *fertilization. Additionally, the effects of the female serum FSH level and sperm quality on the clinical outcome of frozen-thawed oocytes were evaluated.

**Methods:**

Oocytes were slowly frozen and thawed in 22 infertile couples with no sperm or insufficient number of sperm on the day of *in vitro *fertilization (IVF). In 9 couples with severe azoospermia or oligoasthenoteratozoospermia frozen-thawed oocytes were fertilized by autologous sperm of bad quality when available (*Group 1*). In 13 couples with non-ejaculation due to psychological stress on the day of classical IVF or severe azoospermia frozen-thawed oocytes were fertilized by autologous or donated sperm of normal quality (*Group 2*). Oocytes were thawed in 23 cycles and microinjected by the autologous or donated sperm, when available. The clinical outcome of intracytoplasmic sperm injection - ICSI (fertilization, blastocyst, and pregnancy rates) was compared to the outcome of fresh oocytes of the same group of patients; additionally, the female serum FSH level and the sperm quality were compared.

**Results:**

In all couples, 70.5% of oocytes survived the freeze-thaw procedure. After ICSI, 61.5% of thawed oocytes were fertilized. Twenty one% of embryos developed to the blastocyst stage. The pregnancy rates per embryo transfer and freeze-thaw cycle were 33.3% and 17.4%, respectively. All pregnancies ended in the birth of a baby without congenital anomalies. In patients with severe azoospermia or oligoasthenoteratozoospermia there was no statistically significant difference in pregnancy rates per cycle obtained by thawed oocytes vs. fresh oocytes in previous ICSI cycles (14.2% vs. 13.6%) but there was a higher proportion of abnormal, non-cleaved or triploid zygotes when frozen-thawed oocytes were microinjected (33.3% vs. 11.8%; P < 0.01). The female serum FSH levels did not affect the survival and fertilization of frozen-thawed oocytes, but in patients with increased serum FSH level no pregnancies were achieved. After the complete freeze-thaw cycle, there was a significantly higher fertilization rate and tendency to higher pregnancy rates per thawing cycle after the microinjection of autologous or donated sperm of normal quality than autologous sperm of poor quality.

**Conclusion:**

The slow oocyte freezing and thawing is a valuable method when no or insufficient number of sperm are available on the day of *in vitro *fertilization. The quality of sperm is an important factor affecting the clinical outcome achieved by frozen-thawed oocytes.

## Background

The slow freezing method is widely used to freeze human oocytes, both for fertility preservation and in routine IVF programmes. After 5 years of storage in liquid nitrogen, oocytes cryopreserved with a slow cooling-rapid thawing protocol can develop *in vitro *to the blastocyst stage and produce a live birth [1].

Clinical results obtained after *in vitro *fertilization of slowly frozen-thawed oocytes are known to be significantly worse than those obtained with fresh oocytes [2,3]. Despite this fact, oocyte cryopreservation provides the pregnancy rate of about 14% per freeze-thaw cycle and should be offered to patients with surplus oocytes in cases of inapplicability of embryo cryopreservation [4]. The success of human oocyte cryopreservation depends on morphological and biophysical factors that could influence oocyte survival after thawing [5]. At slow freezing, oocytes are frozen with a low concentration of cryoprotectant and may therefore result in a small amount of intra- and extracellular-ice crystal formation, which can damage them. It has been confirmed that slow freezing damages some fine structures of the oocyte, including the meiotic spindle and the zona pellucida. Both the meiotic spindle and the zona pellucida undergo significant changes during slow freezing procedure [6]. The meiotic spindle becomes thinner and structurally less organized (lower retardance), whereas the zona pellucida becomes thicker and its inner layer looses structural organization, as revealed by polarized light microscopy [6].

Little is known about the factors affecting the clinical outcome after using frozen-thawed oocytes in *in vitro *fertilization programme. Morphological changes of the oocyte during freeze-thaw procedure seem to be unrelated to the patient's age or body mass index, but zona pellucida variations in thickness and retardance are significantly related to ovarian responsiveness; patients with a higher response to gonadotrophins produce oocytes that are better able to preserve their characteristics after freezing-thawing [6].

The aim of this retrospective study was to explore the role of oocyte cryopreservation in the group of patients with no available sperm on the day of *in vitro *fertilization; additionally, the effect of the female serum FSH levels and sperm quality on the clinical outcome of frozen-thawed oocytes were evaluated.

## Methods

In this retrospective study, mature (metaphase II) oocytes were cryopreserved in couples with no sperm or insufficient number of sperm to fertilize oocytes on the day of *in vitro *fertilization, to be fertilized when sperm available. In *Group 1 *couples frozen-thawed oocytes were fertilized by autologous sperm of bad quality when available. Their oocytes were cryopreserved, because there were no sperm due to severe azoospermia or oligoasthenoteratozoospermia. In severe azoospermia there was no or insufficient number of sperm in frozen-thawed testicular tissue cryopreserved at the time of a previous diagnostic biopsy. These patients were diagnosed with Sertoli Cell Only Syndrome (SCOS) with focal spermatogenesis or with severe hypospermatogenesis. In patients with severe oligoasthenoteratozoospermia there were no sperm in 2 subsequent ejaculates. In *Group II *couples frozen-thawed oocytes were fertilized by autologous or donated sperm of normal quality. In these couples it was not possible to obtain sperm for fresh oocyte fertilization on the day of *in vitro *fertilization because of severe psychological stress of normospermic partners or severe azoospermia. The semen was considered normal at the concentration of > 20 million sperm per milliliter and > 50% of motile sperm according to the WHO criteria, and at > 14% of morphologically normal sperm according to Kruger's strict criteria. Cryopreserved oocytes were thawed and fertilized when sperm were available. In couples of *Group 1*, thawed oocytes were microinjected by autologous sperm after improvement of the semen quality or testicular biopsy. In couples of *Group 2*, thawed oocytes were microinjected by the fresh autologous sperm of normal quality obtained on the day of oocyte thawing in couples with a normospermic partner or by frozen-thawed donated sperm of normal quality in couples with severe azoospermia. Eeach sperm donation cycle was approved by the Slovenian Assisted Reproduction Authority. This research was approved by the National Medical Ethics Committee; participants agreed to participate in the study by signing an informed consent form.

### *Ovarian stimulation*

For controlled ovarian hyperstimulation we used recombinant FSH (Follitropin alfa) (rFSH) (Gonal F; Industria Farmaceutica Serono S.p.A, Bari, Italy) in combination with GnRH agonist buserelin (Suprefact; Hoechst AG, Frankfurt/Main, Germany).

In the GnRH agonist protocol ovarian stimulation was performed using GnRH agonist buserelin administered from day 22 of the cycle in a daily dose of 0.6 ml (600 pg) s.c. After 14 days, pituitary desensitization was checked by E_2 _determination and B-mode ultrasound scan. Once the criteria for desensitization were fulfilled (E_2 _≤ 0.05 nmol/l, follicles ≤ 5 mm in diameter and endometrial thickness ≤ 5 mm), ovarian stimulation with a daily dose of 225 IU rFSH was started. GnRH agonist administration was continued until HCG administration. HCG (Pregnyl; N.V. Organon, Oss, The Netherlands) in a dose of 10,000 IU was administered when 3 or more follicles reached a diameter of 18 mm. Oocyte retrieval was performed 34-36 h after HCG administration.

### Slow oocyte freezing

Before freezing, oocytes were denuded in hyaluronidase (SynVitroHydase, Origio, Denmark) to evaluate their maturity, and washed 3 times in Flushing Medium (Origio, Denmark). Up to 2 hours after ultrasound-guided retrieval, mature - metaphase II oocytes with extruded polar body were frozen in OocyteFreeze Media (Origio, Denmark). They were washed in PBS and then incubated in the *Freezing solution 1*: 1.5 M propanediol in PBS for 15 minutes at room temperature and in the *Freezing solution II*: 1.5 M propanediol and 0.3 M sucrose in PBS for 15 minutes at room temperature. All PBS included human serum albumin and alpha/beta globulins. At most 2 oocytes were transferred into one 0.3 ml CBS ™ high-security straw (BioSystem, France) with weighted 40 mm identification rod (BioSystem, France). Then, they were cooled in the Air Liquide Machine (France) in liquid nitrogen vapour using the following programme: from 20°C to -8°C at 2°C/min, from -8°C to -30°C at 0.3°C/min, and from -30°C to -150°C at 50°C/min. At -8°C a manual seeding was performed. After cooling, the straws with oocytes were stored in liquid nitrogen (-196°C) until use.

### Rapid oocyte thawing

When used, oocytes were warmed for 40 seconds at the room temperature. They were subsequently transferred into 4 thawing OocyteThaw solutions (Origio, Denmark) - *Thawing solution I*: 1.5M propanediol and 0.2 M sucrose in PBS for 5 min at room temperature, *Thawing solution II*: 0.5 M propanediol and 0.2 M sucrose in PBS for 5 min at room temperature, *Thawing solution III*: 0.2 M sucrose for 10 min at room temperature, *Thawing solution IV*: PBS for 10 min at room temperature, and placed in a CO_2_-incubator for 10 minutes. All PBS included human serum albumin and alpha/beta globulins. Then oocytes were transferred into the fresh, pre-incubated Universal IVF Medium (Origio, Denmark) and incubated in a CO_2_-incubator at 6% CO_2 _in air before microinjection. Oocytes were considered to have survived, if they had unchanged morphology, normal-bright cytoplasm, and zona pellucida of normal appearance. Degenerated oocytes had abnormal shape, damaged zona pellucida, and brown cytoplasm.

### ICSI

Each thawed oocyte was put into a droplet of Sperm Preparation Medium (Origio, Denmark) under paraffin oil (Origio, Denmark). Partner's semen, testicular tissue or frozen-thawed donated sperm was prepared by the density gradient centrifugation on a concentration gradient (100% vs. 40%) of PureSperm (NidaCon, Sweden) and washing in Sperm Preparation Medium (Origio, Denmark). The pellet was used to perform 'swim-up' technique. Up to 2 hours after thawing, the microinjection of oocytes was performed without using the PVP as described elsewhere [7]. After microinjection, oocytes were washed and transferred into the Universal IVF Medium.

### Fertilization, embryo culture and transfer

The next day, 20 to 24 hours after microinjection the oocyte fertilization was checked for observation of the number of pronuclei and polar bodies. Normal diploid zygotes had 2 pronuclei and 2 polar bodies. Triploid and not-cleaved zygotes were not used for transfers.

### Embryo culture

All embryos were cultured in BlastAssist sequential media (Origio, Denmark) to the blastocyst stage. On days 2 and 3 they were cultured in M1 medium and on days 4 and 5 in M2 medium. On day 5 embryos were classified according to their developmental stage into blastocysts, expressing the inner cell mass, trophectoderm, and blastocoele cavity, compacted morulae or arrested embryos. At most 2 embryos at the blastocyst or morula stage were transferred into the uterus by the TDT catheter set (CCD, Neuilly, France). One blastocyst was transferred in couples with only one embryo developed to the blastocyst stage; when more embryos developed to the blastocyst stage, two blastocysts were transferred into the uterus. Embryos were transfered in a natural or modified natural cycle, as described elsewhere [8,9]. Supernumerary blastocysts were cryopreserved on day 5 or 6 by slow freezing program [10].

### Pregnancy

Biochemical pregnancy was confirmed by the positive serum beta-HCG test 15 days after the embryo transfer, and clinical pregnancy by ultrasound scan of gestational sac and embryo heart beats 14 days after the positive beta-HCG test. The data on the live births were provided by the Unit of Obstetrics of our department. The data about the congenital anomalies of children born after the fertilization of frozen-thawed oocytes were retrieved from the medical records of the Unit of Pediatrics which performs the systematic follow-up of children born after assisted reproduction methods.

### Statistics

The correlation between the female serum FSH levels (normal: < 10 IU/L), and semen quality and the clinical outcome after using frozen-thawed oocytes was evaluated by the Pearson and Spearman correlation coefficients. Differences between the groups were evaluated by the Chi-Square test. Statistical significance was set at P < 0.05.

## Results

The mean duration of frozen oocyte storage until thawing was 1 year (min. 0.3 year - max. 2 years). In 23 thawing cycles in 22 couples with the mean female age 31.7 years (range 27 - 40) 70.5% of oocytes survived the freeze-thaw procedure (Table 1). After ICSI, 61.5% of thawed oocytes were fertilized and 21% of embryos developed to the blastocyst stage (Table 1). In 12 (52%) thawing cycles embryos were transferred into the uterus and 4 pregnancies were achieved, yielding a 33.3% pregnancy rate per embryo transfer and a 17.4% pregnancy rate per freeze-thaw cycle, respectively. As shown in Table 1, there was a tendency for higher pregnancy rates per embryo transfer and per thawing cycle in *Group 2 *couples when compared to *Group 1 *couples (42.8% and 23.1% vs. 20% and 11.1%, respectively). All pregnancies were singleton and ended in births of babies with no congenital anomalies.

**Table 1:** Clinical outcome of oocyte freezing and thawing in patients with no sperm or insufficient number of sperm on the day of *in vitro *fertilization

	*Group 1 * Frozen oocytes to be fertilized with autologous sperm of poor quality in severe azoospermia or oligoasthenoteratozoospermia	*Group 2 * Frozen oocytes to be fertilized with autologous or donated sperm of normal quality	TOTAL
**OOCYTE FREEZING**			
Patients	9	13	22
Mean female age (years)	31.3 (range 28-36)	31.9 (range 27-40)	31.7 (range 27 - 40)
Female indications of infertility	4 (44.4%)	10 (76.9%)	14
Freezing cycles	9	14	23
Frozen metaphase II oocytes	83	83	166
Mean number of frozen oocytes per patient	9.2 (min. 4-max. 14)	6.4 (min. 4-max. 12)	7.2 (min. 4 - max. 14)
**OOCYTE THAWING**			
Patients	9	13	22
Thawing cycles	10	13	23
Thawed metaphase II oocytes	83	83	166
Thawed metaphase II oocytes per cycle	8.3 (min. 4-max. 14)	6.4 (min. 4-max. 12)	7.2 (min. 4 - max. 14)
Thawed metaphase II oocytes per patient	9.2 (min. 4-max.14)	6.4 (min. 4-max. 12)	7.5 (min. 4 - max. 14)
Survived oocytes	56 (67.5%)	61 (73.5%)	117 (70.5%)
Fertilized oocytes after ICSI	38 (40.9%)	34 (55.7%)	72 (61.5%)
Embryos	23	34	57
Blastocysts	4	8	12
Blastocyst rate	17.4%	23.5%	21.0%
Embryo transfers (ET)	5	7	12
Double embryo transfers	4	7	11
Pregnancies	1	3	4
Clinical pregnancy rate per ET	20.0%	42.8%	33.3%
Clinical pregnancy rate per thawing cycle	11.1%	23.1%	17.4%
Birth of a child	1	3	4

All differences between the two groups were not statistically significant.

As shown in Table 2, in patients with severe azoospermia or oligoasthenoteratozoospermia (*Group 1*) there were no statistically significant differences in fertilization, blastocyst, and clinical pregnancy rates per cycle obtained by frozen-thawed oocytes vs. fresh oocytes in previous ICSI cycles performed over the time period from 0.3 year to 2.3 years before oocyte thawing (67.1% vs. 50.0%, 23.5 vs. 25.6%, and 14.2% vs. 13.6%, respectively). However, after the microinjection of frozen-thawed oocytes, there was a significantly higher proportion of non-cleaved or triploid zygotes in comparison with fresh oocytes (33.3% vs. 11.8%; P < 0.01). In 2 from 14 (14.3%) oocyte thawing cycles patients did not arrive to the embryo transfer because of non-cleaved or triploid zygotes, whereas in the previous cycles with fresh oocytes none of patients haven't arrive to the embryo transfer because of non-cleaved or triploid zygotes.

**Table 2:** Clinical outcome of ICSI using frozen-thawed oocytes in comparison with fresh oocytes in previous ICSI cycles in patients with severe azoospermia or oligoasthenoteratozoospermia

	ICSI in patients with severe azoospermia or oligoasthenoteratozoospermia	
	Fresh oocytes	Frozen-thawed oocytes
Patients	13	13
ICSI cycles	22	14
Injected oocytes	171	111
Fertilized oocytes	85 (50.0%)	51 (67.1%)
Non-cleaved or triploid zygotes	10 (11.8%)*	17 (33.3%)*
Embryos	78	34
Blastocysts	20	8
Blastocyst rate	25.6%	23.5%
Embryo transfers (ET)	18	7
Pregnancies	3	2
Clinical pregnancy rate per ET	17.0%	28.6%
Clinical pregnancy rate per cycle	13.6%	14.2%
Birth of a child	3	2

*Statistically significant difference as evaluated by the Chi-Square test at *P < 0.01*.

The mean serum FSH level in female patients was 7.4 (min. 4.1-max. 18.0) IU/L. In 18 oocyte thawing cycles performed in 18 patients with a normal serum FSH level there was approximately the same oocyte survival rate than in 4 thawing cycles performed in 3 patients with increased FSH level (70.4% vs. 67.0%). In patients with normal and increased serum FSH levels the same percentage (40%) of thawed oocytes were normally fertilized and developed into the embryos. In patients with normal serum FSH levels 4 (22,2%) pregnancies were achieved, whereas none of patients with the increased serum FSH levels became pregnant.

When in *Group 2 *patients with severe azoospermia or oligoasthenoteratozoospermia frozen-thawed oocytes were microinjected with frozen-thawed donor sperm of normal quality, the fertilization rate was significantly higher than after the microinjection of autologous sperm of poor quality (72.2% vs. 40.9%; P < 0.05) *in Group 1 *patients, as shown in Table 3. There was also a tendency to a higher clinical pregnancy rate after the microinjection of normal sperm than after the microinjection of sperm of poor quality (25.0% vs. 11.1%), as shown in Table 3. Similarly, in *Group 2 *patients the clinical pregnancy rate per freeze-thaw cycle achieved by the microinjection of autologous sperm of normal quality was comparable to the microinjection of donated sperm of normal quality (22.2% vs. 25.0%).

**Table 3:** Clinical outcome of injection of frozen-thawed donor sperm of normal quality vs. autologous fresh sperm of poor quality into frozen-thawed oocytes

	ICSI of frozen-thawed oocytes in couples with severe azoospermia or oligoasthenoteratozoospermia	
	DONOR SPERM (normal quality)	AUTOLOGOUS EJACULATED OR TESTICULAR SPERM (poor quality)
Patients	4	9
ICSI cycles	4	10
Mean female age (years)	31.2 (range 28 - 38)	31.1 (range 28 - 37)
Injected oocytes	18	93
Fertilized oocytes	13 (72.2%)*	38 (40.9%)*
Embryos	11	23
Blastocysts	4	4
Blastocyst rate	36.4%	17.4%
Embryo transfers (ET)	2	5
Pregnancies	1	1
Clinical pregnancy rate per ET	50.0%	20.0%
Clinical pregnancy rate per thawing cycle	25.0%	11.1%
Birth of a child	1	1

*Statistically significant difference as evaluated by the Chi-Square test at *P < 0.05*.

After ICSI of frozen-thawed oocytes, 4 blastocysts were cryopreserved in 2 patients: in one patient 1 blastocyst and in another one 3 blastocysts. After thawing, all blastocysts (100%) were survived and were completely non-damaged according to the morphological criteria. All thawed blastocysts were transferred into the uterus, but both patients did not become pregnant.

## Discussion

The results of this study show that the slow oocyte freezing and thawing is a valuable method in patients with no or insufficient number of sperm available on the day of *in vitro *fertilization.

In this study, mature oocytes were frozen for up to 2 hours after the ultrasound-guided oocyte retrieval. In line with other researchers, mature oocytes which were slowly frozen within this time period yielded a higher amount of good quality embryos after ICSI than oocytes, slowly frozen beyond that time period [11]. The time at which oocyte cryopreservation is performed plays a key role in the clinical outcome of frozen-thawed oocytes and should be performed at less than 40 hours after HCG administration [12]. Cryoprotectant 1,2-propanediol and increased concentration 0.3 M sucrose were used to optimize the oocyte slow freezing procedure [5].

In all patients included into this study, an overall 33.3% pregnancy rate per embryo transfer and a 17.4% pregnancy rate per thaw cycle were obtained. These pregnancy rates could be compared to the results of other groups performing slow oocyte freezing [4,13,14]. Albani *et al. *reported that the pregnancy and implantation rates achieved by slowly frozen and thawed oocytes steadily improved from respectively 6.7% and 2.4% in 2001 to 15% and 8.2% in 2007 [15]. The results of this study showed that the results after slow oocyte freezing and thawing were better after fertilization of thawed oocytes by autologous or donated sperm of normal quality than after fertilization by autologous sperm of bad quality. Pregnancy rates achieved by fertilization of thawed oocytes by sperm of normal quality were higher than results reported by other groups [4,13-15]: 23.1% per thawing cycles by autologous sperm and 25,0% per thawing cycles by donated sperm thus indicating that normal sperm quality in male partner or decision for donated sperm of normal quality is an additional indication for slow oocyte freezing when no sperm available on the day of *in vitro *fertilization. The general data of this study - pregnancy rates achieved by the frozen-thawed oocytes allow us to counsel future patients in our clinical practice who face a similar situation of not being able to collect sperm on the day of oocyte retrieval and *in vitro *fertilization.

In the group of men with severe azoospermia or oligoasthenoteratozoospermia, there was no difference in the clinical outcome of frozen-thawed oocytes inseminated by ICSI when compared to their previous ICSI attempts with fresh oocytes. The fertilization and blastocyst rates did not differ between ICSI cycles using fresh and frozen-thawed oocytes. But, there was a significantly higher proportion of non-cleaved or triploid zygotes after insemination of frozen-thawed oocytes by ICSI. This might be related to the disturbances of oocyte meiotic spindle and some other disturbances during slow freezing and thawing procedures as confirmed by several basic studies in animal models [16-18], and also in humans [19-22].

In spite of increased percentage of non-cleaved or triploid zygotes, the normal blastocyst rate, a high pregnancy rate per embryo transfer (33.3%), and births of babies with no congenital anomalies in this study showed that the negative effect of slow oocyte freezing and thawing might be expressed at the stage of zygote (especially non-cleaved), and not later, at the embryo stage.

The negative effect of increasing female age on the oocyte and embryo quality has been well established [23-27], but it should be emphasized that the mean age of women in the present study was 31.7 years (range 27 - 40), lower than the mean age of our *in vitro *fertilization program (34.7 years). Molinari *et al. *have found that the morphological changes of oocytes during the freeze-thaw procedure seem to be unrelated to the patient's age [6]. But, there are no data available in the literature on clinical outcomes of slow oocyte freezing and thawing in older women. We cannot make any conclusions about the affect of female age on the outcome of oocyte freeze-thawing in this study, because included women were too young. In terms of serum FSH level it should be noted that most women included in this study had normal levels of serum FSH levels and had a normal ovarian response to the hormonal stimulation, thus providing the possibility to freeze the mean number of 7.2 mature oocytes per a patient. In women with normal and increased serum FSH levels the oocyte survival and fertilization rates after freeze-thawing procedures did not differ significantly. Although in all patients blastocysts or morulae were transferred, 4 pregnancies were achieved in women with normal serum FSH levels, while none of women with increased serum FSH levels became pregnant. This might reflect worse quality of oocytes and embryos in patients with increased serum FSH levels.

On the other hand we found that the sperm quality affected the outcome of ICSI using frozen-thawed oocytes. The results obtained by frozen-thawed oocytes microinjected with autologous or donated sperm of normal quality were better than after the microinjection of autologous sperm of poor quality. It is generally known that in infertile men, sperm of poor quality are related to a higher level of impaired DNA integrity (i.e., DNA fragmentation), which might result in a lower fertilization potential, worse embryo development, and spontaneous abortion [28,29].

All neonates, born in this study were normal and did not have any congenital malformations. Although short term neonatal outcomes were evaluated in this study, we do not have long term infant outcomes for these oocyte thawing cycles. Previous studies suggested that the incidence of congenital malformations in neonates after fertilization of frozen-thawed oocytes was not increased [30]; there was any difference in the occurrence of congenital malformations between the live borns after oocyte slow freezing and naturally conceived infants. The best way to study long-term outcomes would be to set up a birth registry which is already in the works [30].

The results of this study showed, that slow oocyte freezing and thawing in infertile couples with no sperm available on the day of *in vitro *fertilization seem to be safe methods, expressing their negative effects at the zygote level and resulting in births of healthy newborns. New method - oocyte vitrification results in high pregnancy rates [31-33], and seems to inflict less damage to spindle integrity and chromosome alignment during oocyte cooling [34,35], although it might pose a problem of due to a very high concentration of potentially toxic cryoprotectant in the vitrification solution. It is known, that high concentrations of cryoprotectants could induce *in vitro *chromosomal damage in eukaryotic cells [36]. Some studies showed that in human oocytes subjected to vitrification some histones were modified: H3K9 methylation and H4K5 acetylation were increased [37]; it was proposed, that both of these histone modifications could be useful markers to monitor epigenetic perturbations induced by various experimental vitrification protocols [37]. Properly controlled follow-up studies of neonatal outcomes, and child long-term follow-up studies for all oocyte cryopreservation techniques are needed and have already been proposed [38].

Slow oocyte freezing and thawing is a valuable method when no or insufficient number of sperm are available on the day of *in vitro *fertilization. The quality of sperm is an important factor affecting the clinical outcome achieved by frozen-thawed oocytes. The results of this study allow us to counsel future patients in our clinical practice who face a similar situation of not being able to collect sperm on the day of oocyte retrieval and *in vitro *fertilization.

## Competing interests

The authors declare that they have no competing interests.

## Authors' contributions

IVK was a leader of the research project, who introduced the program of oocyte cryopreservation into the IVF Lab practice. She made all the calculations, evaluated the clinical outcome, and drafted the manuscript. LBK practically performed most of the oocyte freeze-thawing procedures. TT and EVB performed the ovarian stimulation and preparation of patients for the transfer of embryos after microinjection of frozen-thawed oocytes. All authors read and approved the final manuscript.
